# Sustained Enlargement in Vagus and Sural Nerve Cross‐Sectional Areas in Fibromyalgia: A Longitudinal Study

**DOI:** 10.1111/jon.70055

**Published:** 2025-06-01

**Authors:** Benedetta Bianchi, Edoardo Cipolletta, Sonia Farah, Fausto Salaffi, Marco Di Carlo

**Affiliations:** ^1^ Rheumatology Unit Università Politecnica delle Marche, Ospedale “Carlo Urbani” Jesi Ancona Italy

**Keywords:** CSA, fibromyalgia, imaging, peripheral nerves, US

## Abstract

**Background and Purpose:**

Fibromyalgia (FM) is a complex condition with unclear pathophysiology. While central sensitization is commonly accepted as the predominant cause of pain symptoms, numerous evidences suggest a role for the peripheral nervous system, particularly small fiber neuropathy. Previous studies have documented that patients with FM show an increased cross‐sectional area (CSA) of some nerves, including the vagus and sural nerves, detectable via ultrasound (US). The purpose of this study is to assess whether the CSA increase persists over time and to investigate potential correlations between nerve dimensions and clinical variables.

**Methods:**

This study involved 32 female patients with FM and 20 healthy controls, both evaluated at baseline and after 24 months. Participants completed clinimetric questionnaires addressing disease severity, neuropathic pain features, and autonomic dysfunction, while US measurements of the vagus and sural nerves' CSA were taken. Differences in CSA variation were assessed with student's t‐test and chi‐square, and the Pearson's correlation coefficient tested relationships between nerve dimensions and clinimetric scores.

**Results:**

CSA values were higher in FM patients compared to controls at both baseline and after 24 months, although no significant differences in CSA changes were found over time. Pearson's correlation revealed some associations between nerve dimensions and clinimetric scores, suggesting potential relationships that require further investigation.

**Conclusions:**

FM patients exhibit persistent increases in the vagus and sural nerves CSAs. Further studies are needed to better understand the clinical significance of these findings and the role of US assessment as a tool for detecting nerve alterations in FM.

## 1 Introduction

Fibromyalgia (FM) is a complex condition characterized by chronic widespread pain and a range of somatic, emotional, and neuropsychological symptoms. The lack of specific biological or instrumental biomarkers has made FM a topic of ongoing debate. Current hypotheses suggest that FM's development may be explained through the bio psychosocial model, which includes genetic predisposition, stressful life events, cognitive‐emotional factors, and pain sensitization processes [1]. Recent studies describe FM as a condition characterized by nociplastic pain, a term used to denote pain resulting from altered nociception without objective evidence of tissue or somatosensory damage. Nociplastic pain is closely linked to central sensitization, a mechanism in which the central nervous system becomes hyper‐responsive to sensory stimuli, leading to pain amplification and hypersensitivity [2, 3].

The involvement of the central nervous system in FM pathogenesis is accepted, though its precise role is still being investigated. Neuroimaging studies reveal that FM patients exhibit reductions in gray matter volume in specific regions. Functional magnetic resonance imaging (MRI) studies have demonstrated increased cortical blood flow in pain‐processing regions of the brain and reduced connectivity within the descending pain‐modulation system. Furthermore, repetitive stimulation of C‐fibers, as seen in chronic pain conditions, may cause apoptosis of inhibitory interneurons, resulting in an increase in pain‐signaling molecules and a reduction in inhibitory modulation. In addition, FM pathogenesis may involve the over activation of microglial cells, mediated by stimulation of the toll‐like receptor 4 (TLR4). Another mechanism contributing to FM symptoms is dysregulation of hormone levels, particularly of those associated with the hypothalamic‐pituitary‐adrenal axis. Additionally, abnormalities in the autonomic nervous system, such as reduced microcirculatory and vasoconstrictive responses, have been observed in FM patients, potentially impairing stress management and contributing to increased pain [4].

Recent discussions have also focused on the possible involvement of the immune system in FM through a process of “neuroinflammation.” This mechanism appears to be mediated by autoantibodies, such as anti‐G protein‐coupled receptor antibodies, which may contribute to the widespread symptoms by damaging small nerve fibers [1].

Damage to small nerve fibers, such as A‐delta and C‐fibers, results in small fiber neuropathy (SFN), a condition associated with disorders like glucose metabolism defects, immune dysregulation, gluten sensitivity, monoclonal gammopathy, vitamin deficiencies, toxin exposure, and cancer. Some FM patients also seem to have undiagnosed or concurrent SFN. Emerging evidence challenges the traditional view by highlighting the potential role of the peripheral nervous system in FM pathogenesis. This peripheral involvement is supported by a meta‐analysis indicating that approximately 49% of FM patients exhibit signs of SFN, as defined by objective tests, challenging the prevailing concept that FM is primarily a central nervous system disorder [5]. A link between obesity, chronic pain, and FM has been established, with symptoms such as dysautonomia and paresthesia potentially pointing to underlying SFN.

Structural abnormalities in C‐fibers and impaired efferent functions may significantly contribute to FM pathogenesis. Neurogenic microvasculopathy, due to small fiber nerve dysfunction, could explain muscle perfusion deficits, deep pain, exercise intolerance, and the cognitive symptoms often referred to as “fibro fog” in FM. An “autoimmune process” involving the release of pain‐related cytokines and other immune mediators might also play a role in distal nerve fiber degeneration [6]. Neurogenic inflammation, particularly involving C‐fibers, appears to be implicated, with inflammatory markers such as IgG, tumor necrosis factor‐alpha, and interleukin‐6 identified in skin biopsies of FM patients. Furthermore, studies have noted differences between FM‐related SFN and idiopathic SFN, including a higher prevalence of ballooned Schwann cells in FM, suggesting distinct underlying mechanisms. An association between obesity, impaired glucose tolerance, and FM has also been found, implying that weight loss and exercise may alleviate neuropathic pain and improve small nerve fiber density [5].

SFN manifests as pain, sensory disturbances, or autonomic dysfunction, and neuropathic pain features are acknowledged by some FM diagnostic tools. For example, the Fibromyalgia Rapid Screening Tool (FiRST) includes items assessing sensations such as pinprick, tingling, and numbness [7]. The diverse clinical manifestations of SFN, coupled with the absence of detectable abnormalities during physical examination, make its diagnosis challenging. In FM patients, symptoms substantially overlap between those with and without confirmed SFN, though dysautonomia and paresthesias may point to underlying peripheral involvement [8]. Screening for SFN is crucial, as it has identifiable and potentially treatable causes [5]. Several diagnostic methods have been proposed for SFN detection. Quantification of intraepidermal nerve fiber density (IENFD) through distal leg skin biopsy is currently the most accurate diagnostic method for SFN [9]. However, this method is invasive, time‐consuming, and expensive. Corneal confocal microscopy, by contrast, offers a non‐invasive, efficient, and highly reproducible means of quantifying nerve fiber density in vivo [10], though its integration into clinical practice is challenging. Quantitative sensory testing, which assesses sensitivity to temperature, pressure, vibration, and electrical stimulation, is limited by tester‐ and environment‐related factors, and the absence of age‐ and sex‐specific threshold values further restricts its clinical applicability [11].

Some studies document a potential role for nerve ultrasonography (US) in suggesting the presence of SFN [12]. A 2015 study reported that patients with SFN diagnosed by reduced IENFD, compared to healthy subjects, exhibit an increased cross‐sectional area (CSA) of the sural nerve [13]. This suggests that morphological changes occur in certain nerves in SFN, which can be identified through a simple, non‐invasive ultrasound (US) examination that is widely accessible to most clinicians.

A previous cross‐sectional study conducted by our group revealed that, compared to healthy subjects, patients with FM show an increased CSA at specific peripheral nerve sites, primarily the sural nerve, the vagus nerve, and the sixth cervical nerve root [14]. However, longitudinal data are lacking; it remains unclear whether the increased CSA persists over time.

Based on these premises, the present 24‐month longitudinal study aims to confirm or not the persistence of increased CSA in the vagus and sural nerves of FM patients compared to healthy controls. An additional objective is to examine correlations between US findings, clinimetric indices of disease severity, neuropathic pain features, and symptoms of autonomic dysfunction.

## 2 Methods

### 2.1 Setting and Study Design

This longitudinal observational study was conducted at a third‐level rheumatology center, the Rheumatology Unit at Università Politecnica delle Marche, “Carlo Urbani” Hospital, Jesi (Ancona), Italy, between October 2021 and June 2024. The Unit has extensive experience in the diagnosis, treatment, and research on FM.

Ethical approval for the study was obtained from the comitato etico unico regione marche (number 1970/AST Ancona). All participants provided written informed consent, and the study adhered to the principles of the 1964 Declaration of Helsinki and its amendments.

### 2.2 Inclusion and Exclusion Criteria

Female patients diagnosed with FM according to the 2016 American College of Rheumatology criteria were consecutively included in the study. Exclusion criteria comprised: diseases potentially associated with SFN, such as diabetes mellitus, uncontrolled endocrinopathies, ongoing neoplasms, chronic viral infections (hepatitis B, hepatitis C, human immunodeficiency virus), connective tissue diseases, vasculitis, sarcoidosis, amyloidosis, Ehlers‐Danlos syndrome, or joint hypermobility syndrome; history of alcoholism or use of neurotoxic drugs; diseases affecting the central or peripheral nervous system (e.g., Alzheimer's disease, Parkinson's disease, motor neuron disease, multiple sclerosis, and spinal lesions); large fiber neuropathy indicated by electrophysiological studies or by clinical assessment (with an examination carried out to detect signs such as loss of joint position and vibration sense, and sensory ataxia); concomitant musculoskeletal diseases that might interfere with clinical evaluation (e.g., chronic inflammatory joint diseases, crystal arthritides, symptomatic osteoarthritis); and history of safenectomy due to potential sural nerve injury.

Healthy female controls without any of the aforementioned conditions were recruited from hospital staff and patients' relatives.

### 2.3 Demographic Variables

Clinical and clinimetric evaluations were conducted by an experienced rheumatologist (FS), with over 30 years of experience in managing FM patients. At both baseline and after 24 months, the following demographic, anthropometric, and clinical data were collected: age, weight, height (in order to calculate body mass index [BMI]), disease duration, and drug treatments.

### 2.4 Clinimetric Assessment

FM severity was assessed using the revised Fibromyalgia Impact Questionnaire (FIQR), which includes 21 questions, each rated on an 11‐point scale from 0 to 10, referring to the past 7 days. It is divided into three domains: the “functional” domain (assessing difficulties in daily activities), the “overall impact” domain (evaluating the impact of FM on the patient's life), and the “symptoms” domain (including questions on memory, tenderness, balance, and sensitivity to environmental stimuli). The total FIQR score is a weighted sum of the previous domains; the maximum score is 100, with higher scores indicating greater disease severity [15].

Neuropathic pain features were assessed using the Douleur Neuropathique four Questions (DN4), which consists of 10 items evaluating neuropathic symptoms and neurological assessments. The first two questions (seven elements) address symptoms like burning pain, painful cold, electric shocks, tingling, pinpricks, numbness, and itching. The last two questions (three elements) involve a physician's assessment of tactile hypoesthesia, pinprick hypoesthesia, and touch‐evoked allodynia. Each element scores 1 if present and 0 if absent, with a total score of 0–10. A score of ≥4 indicates the presence of neuropathic pain [16].

Autonomic symptoms were evaluated with the Composite Autonomic Symptom Score 31 item (COMPASS‐31), comprising 31 questions across domains assessing various autonomic nervous system functions: orthostatic intolerance, vasomotor, secretomotor, gastrointestinal, bladder, and pupillomotor. Each question is scored based on symptom presence, severity, frequency, and time course, with higher total scores (ranging from 0 to 100) indicating more severe autonomic symptoms [17].

Pain and associated symptoms were investigated through the Widespread Pain Index (WPI) and the Symptom Severity Scale (SSS). The WPI assesses 19 areas of the body; patients are asked to mark the areas where they have experienced pain most intensely over the past seven days. The number of marked areas corresponds to the final score, which ranges from 0 to 19. For the determination of the SSS, symptoms such as fatigue, unrefreshing sleep, cognitive disturbances, and somatic symptoms are evaluated. Each symptom is assigned a score between 0 and 3, with increasing severity. The final score, ranging from 0 to 12, is obtained by a simple sum.

### 2.5 Nerve US Examination

Nerve US examinations were conducted both at baseline and after 24 months by a rheumatologist with over 10 years of experience in musculoskeletal and nerve US (MDC). The sonographer was blinded to the clinical and clinimetric evaluations. A MyLab Class C (Esaote S.p.A., Genoa, Italy) US machine with a 6–18 MHz multifrequency linear probe for the vagus nerve and a 10–22 MHz probe for the sural nerve was used.

For the purposes of this study, an US examination was conducted exclusively on the vagus nerve and the sural nerve, which are the sites where a difference in CSA has been demonstrated between patients with FM and healthy subjects [14]. The sixth cervical nerve root, although it is one of the sites where CSA has been shown to be significantly increased in FM patients compared to controls, was not considered due to the feasibility of the US examination, as it is technically more difficult to scan and potentially influenced by anatomical variables such as a short neck and obesity.

**FIGURE 1:**
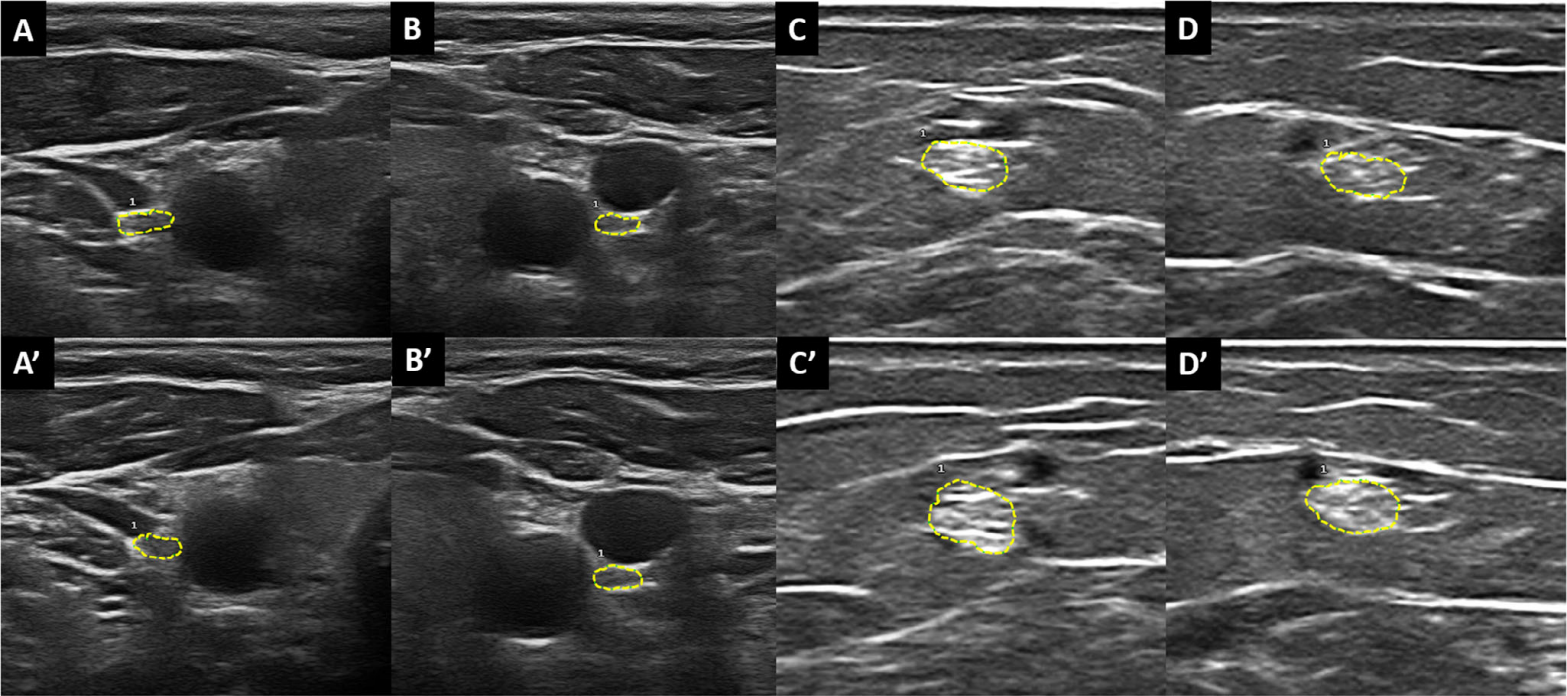
Ultrasound scans of nerves in a patient with fibromyalgia, assessed at baseline (A, B, C, and D) and at the 24‐month follow‐up (A’, B’, C’, and D’). Panels A and A’ depict the right vagus nerve, B and B’ the left vagus nerve, C and C’ the right sural nerve, and D and D’ the left sural nerve. Compared to a healthy subject, all cross‐sectional areas (CSA) are increased, measuring 5 mm^2^ for all vagus nerve assessments at both baseline and follow‐up, 8 squared millimeters (mm^2^) for the right sural nerve at both time points, 6 mm^2^ for the left sural nerve at baseline, and 7 mm^2^ at follow‐up. The CSA regions are delineated with fine yellow dashed lines.

**FIGURE 2:**
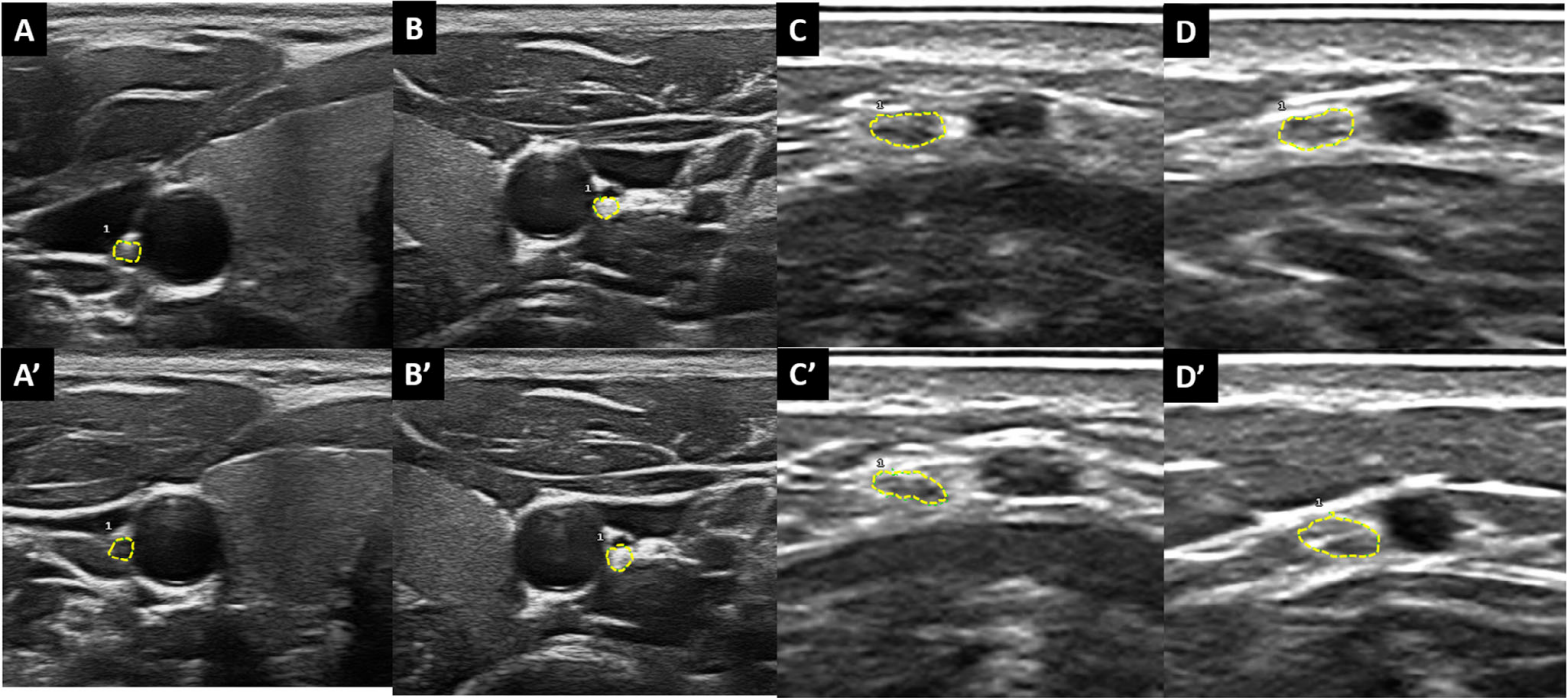
Ultrasound scans of nerves in a healthy subject, assessed at baseline (A, B, C, and D) and at the 24‐month follow‐up (A’, B’, C’, and D’). Panels A and A’ depict the right vagus nerve, B and B’ the left vagus nerve, C and C’ the right sural nerve, and D and D’ the left sural nerve. All measured cross‐sectional area (CSA) values are 2 squared millimeters. The CSA regions are delineated with fine yellow dashed lines.

The nerve CSA was measured in US frozen images by freehand technique (tracing a line inside the hyperechoic margin of the nerve), repeating the measure three times and calculating the mean (expressed in mm^2^). The CSA of the vagus nerve was scanned on the midcervical plane (lateral to the thyroid cartilage), beneath the carotid artery and the internal jugular vein; here, the vagus nerve lies between the artery and vein. In a transversal view, nerves appear as small, round hypoechoic elements, sometimes having a honeycomb structure [18]. The CSA of the sural nerve was measured on the posterolateral side of the calf, about 14 cm proximal to the lateral malleolus [19]. Each nerve was investigated bilaterally. Examples of US images in patients and healthy subjects are provided in Figure 1 and Figure 2.

### 2.6 Statistical Analysis

Descriptive statistics, including the mean, standard deviation (SD), and median, were reported for demographic and clinimetric data.

All analyses conducted on the CSA of the vagus and sural nerves were considered as the sum of bilateral values. The normality of the data distribution was confirmed using the Shapiro‐Wilk test and visual inspection of histograms.

The paired t‐test and chi‐square test were employed to compare the CSA of the vagus and sural nerves at baseline and after 24 months. The Pearson test was used to correlate the US findings in the FM cohort (both at baseline and after 24 months) with main clinimetric variables (FIQR, COMPASS‐31, DN4, WPI, and SSS).

Statistical analysis was conducted using SPSS software (version 25.0; SPSS Inc., Chicago, IL, USA, https://www.ibm.com/spss).

## 3 Results

### 3.1 Demographic Data and Clinimetric Assessment at Baseline

Thirty‐two female FM patients (mean age 54.8 ± 9.7 years, BMI 26.9 ± 5.8 kg/m^2^) and 20 healthy female controls (mean age 53.9 ± 7.6 years, BMI 23.9 ± 5.4 kg/m^2^) were enrolled in the study. As there were no significant differences in BMI (*p* = 0.78) or age (*p* = 0.048) between the groups, they were considered homogeneous.

Regarding pharmacological treatment, 11 patients received duloxetine (34.4%), 13 pregabalin (40.6%), 3 benzodiazepines (9.4%) and 7 other antidepressants (21.9%). Additionally, 10 were treated with L‐acetylcarnitine (31.2%) and 3 with palmitoylethanolamide (9.4%).

At baseline, FM patients had a mean FIQR score of 67.5 (± 16.6), a DN4 of 5.3 (± 2.5), a COMPASS‐31 of 43 (± 17.5), a WPI of 11.5 (± 4.0), and a SSS of 8.6 (± 2.8). Table 1 summarizes the main clinical and demographic characteristics.

**TABLE 1:** Demographic and clinimetric data of the population sample at baseline (32 patients).

	Mean	SD	Median	IQR
BMI (kg/m^2^)	26.9	5.8	25.0	23.0 – 30.3
Age (years)	54.8	9.7	54.0	48.5 – 63.0
WPI	11.5	4.0	12.0	9.0 – 13.0
SSS	8.6	2.8	9.0	7.0–11.0
FIQR physical function	59.9	15.5	60.0	52.8–69.8
FIQR symptoms	13.5	4.4	13.5	10.0–17.3
FIQR overall impact	68.2	17.0	68.0	60.3–81.0
FIQR total	67.5	16.6	66.7	61.2–79.4
COMPASS‐31 total	43.0	17.5	42.8	29.9–54.7
DN4 patient‐reported	3.9	2.2	4.0	1.0–6.0
DN4 objective assessment	1.5	1.0	2.0	1.0–2.0
DN4 total	5.3	2.5	6.0	3.0–7.0

Abbreviations: BMI, body mass index; COMPASS‐31, Composite Autonomic Symptom Score 31 item; DN4, Douleur Neuropathique four questions; FIQR, revised Fibromyalgia Impact Questionnaire; IQR, interquartile range; kg, kilogram; m^2^, square meter; SD, standard deviation; SSS, Symptom Severity Scale; WPI, Widespread Pain Index.

### 3.2 US Findings

**FIGURE 3:**
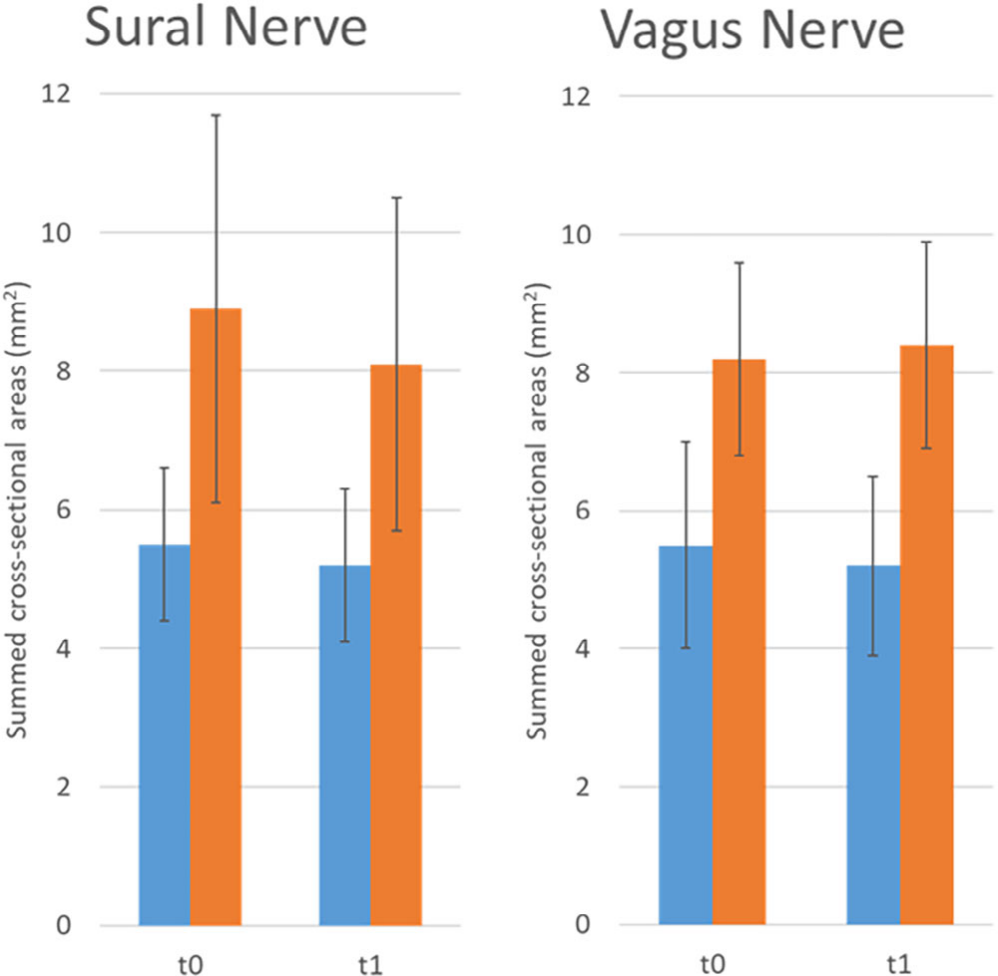
Histogram depicting nerve dimensions (sum of right and left cross‐sectional areas, espressed in square millimeters [mm^2^]) at baseline (t0) and after 24 months (t1). Left panel illustrates sural nerve values, with blue bars representing controls and orange bars representing patients. Right panel shows vagus nerve values, with the same color scheme. Vertical black lines indicate standard deviation.

The CSA values for both patients and healthy controls at baseline and after 24 months (expressed as the means and *SD* of the summed CSA) are summarized in Table 2, including delta values representing the change over time. In the FM group, the summed CSA of the sural nerve moved from 8.9 (±2.8) mm^2^ at baseline to 8.1 (±2.4) mm^2^ after 24 months, while the vagus nerve showed a slight increase from 8.2 (±1.4) mm^2^ to 8.4 (±1.5) mm^2^. In healthy controls, the sural nerve CSA shifted from 5.5 (±1.1) mm^2^ to 5.2 (±1.1) mm^2^, while the vagus nerve CSA remained relatively stable (5.7 ±1.1 mm^2^ to 5.8 ±1.3 mm^2^). At baseline, FM patients had significantly larger CSA values for both the sural and vagus nerves compared with healthy controls (*p* < 0.001), and this difference persisted after 24 months (*p* < 0.001). Figure 3 shows differences between the two groups.

**TABLE 2:** Summed cross‐sectional area values (expressed as means and standard deviations of the sum of right and left nerves) at baseline, final values, and delta values.

CSAs	Controls (*n* = 20)	Patients (*n* = 32)	Total (*n* = 52)	*p*‐value
Sural nerve CSA (mm^2^) baseline	5.5 (1.1)	8.9 (2.8)	7.6 (2.8)	<0.001
Vagus nerve CSA (mm^2^) baseline	5.7 (1.5)	8.2 (1.4)	7.2 (1.9)	<0.001
Sural nerve CSA (mm^2^) final	5.2 (1.1)	8.1 (2.4)	7.0 (2.4)	<0.001
Vagus nerve CSA (mm^2^) final	5.8 (1.3)	8.4 (1.5)	7.4 (1.9)	<0.001
Sural nerve CSA delta values (mm^2^)	−0.3 (1.2)	−0.8 (2.1)	−0.6 (1.8)	0.289
Vagus nerve CSA delta values (mm^2^)	0.1 (1.6)	0.3 (1.5)	0.2 (1.5)	0.765

Abbreviations: CSA, cross‐sectional area; mm^2^, square millimeter.; *n*, sample size.

### 3.3 Correlation Analyses

Correlation analyses were performed between the summed CSA of the sural and vagus nerves (at baseline, after 24 months, and in terms of delta values) and various clinimetric scores, including WPI, SSS, FIQR, COMPASS‐31, and DN4, all assessed at baseline, after 24 months, and as delta values. Among the indices analyzed, the most significant correlations with nerve dimensions were observed for the COMPASS‐31 and DN4 scores. COMPASS‐31 was found to be linked to sural nerve dimensions, with baseline COMPASS‐31 correlating with the final CSA (*p* = 0.013). In contrast, patients with higher DN4 scores, both at baseline and after 24 months, exhibited a greater increase in vagus nerve size (*p* = 0.004 and *p* = 0.002, respectively), resulting in higher final CSA values (*p* = 0.014 and *p* = 0.017, respectively).

Detailed correlation coefficients (*r*) and *p*‐values for each clinimetric score, in relation to the sural and vagus nerve CSA at baseline, after 24 months, and delta values, are provided in Table 3.

**TABLE 3:** Correlations between summed cross‐sectional area (at baseline, final values, and delta values) and clinimetric indices (at baseline, final values, and delta values).

			Baseline		Final		Delta values	
			Sural nerve	Vagus nerve	Sural nerve	Vagus nerve	Sural nerve	Vagus nerve
WPI	T0	r	0.180	−0.075	0.172	0.123	−0.046	0.194
		p	0.323	0.685	0.347	0.503	0.805	0.287
	T1	r	0.208	0.253	0.169	−0.060	−0.087	−0.298
		p	0.253	0.163	0.355	0.742	0.636	0.098
	Delta	r	0.058	0.360	0.022	−0.190	−0.054	−0.529
		p	0.752	0.043^*^	0.904	0.298	0.770	0.002^*^
SSS	T0	r	0.254	−0.008	0.226	−0.018	−0.083	−0.011
		p	0.161	0.965	0.214	0.922	0.650	0.954
	T1	r	0.092	0.069	−0.007	0.086	−0.134	0.022
		p	0.618	0.707	0.969	0.639	0.463	0.903
	Delta	r	−0.212	0.079	−0.277	0.107	−0.034	0.035
		p	0.244	0.669	0.125	0.559	0.852	0.850
FIQR	T0	r	0.176	0.244	0.186	0.061	−0.023	−0.166
		p	0.335	0.179	0.307	0.740	0.901	0.364
	T1	r	0.219	0.174	0.123	−0.193	−0.156	−0.358
		p	0.229	0.341	0.503	0.289	0.394	0.044^*^
	Delta	r	0.045	−0.082	−0.074	−0.286	−0.149	−0.213
		p	0.808	0.656	0.687	0.112	0.416	0.242
COMPASS‐31	T0	r	0.343	0.109	0.390	−0.105	−0.012	−0.208
		p	0.055	0.553	0.027	0.568	0.950	0.254
	T1	r	0.004	0.187	0.132	−0.259	0.150	−0.437
		p	0.985	0.305	0.473	0.153	0.411	0.013^*^
	Delta	r	−0.388	0.051	−0.323	−0.122	0.154	−0.171
		p	0.028^*^	0.784	0.072	0.506	0.401	0.351
DN4	T0	r	0.068	0.068	−0.040	−0.431	−0.141	−0.499
		p	0.713	0.712	0.826	0.014^*^	0.443	0.004^*^
	T1	r	0.201	0.113	0.089	−0.420	−0.171	−0.530
		p	0.271	0.539	0.628	0.017^*^	0.348	0.002^*^
	Delta	r	0.173	0.064	0.155	−0.038	−0.055	−0.098

Abbreviations: COMPASS‐31, Composite Autonomic Symptom Score 31 item; delta, difference between final and itial values; DN4, Douleur Neuropathique four questions; FIQR, revised Fibromyalgia Impact Questionnaire; T0, baseline; SSS, Symptom Severity Scale; T1, 24 months after baseline; WPI, Widespread Pain Index.

* : significant correlations.

## 4 Discussion

This study documented that, over a 24‐month period, the characteristic morphological alterations of specific nerves—namely the sural nerve in the lateral portion of the leg and the vagus nerve at the neck level—assessed via US in patients with FM compared to healthy subjects, remain stable over time.

In a recent review investigating SFN in FM, all skin biopsy studies consistently reported a reduction in IENFD in FM patients compared to healthy controls. Biopsies from different sites on the leg revealed a uniform decrease in IENFD, indicating SFN without a distal gradient, which may suggest ganglionopathy. Additionally, some studies reported abnormalities in nerve fiber regeneration and small blood vessel function. Schwann cell ballooning and peripheral axonal abnormalities, including reduced axon size, were highlighted in FM patients. Corneal confocal microscopy studies further confirmed peripheral nervous system involvement, reporting decreased nerve fiber length, density, and branching, along with alterations in corneal innervation and Langerhans cell distribution. Microneurography studies identified abnormal somatic small fiber function, including spontaneous activity and increased sensitivity to mechanical stimuli.

Recent evidence suggests that SFN may contribute to the pathogenesis of autonomic disturbances, such as orthostatic intolerance, flushing, thermal intolerance, and digestive and sudomotor abnormalities. Studies utilizing heart rate variability (HRV) analysis have reported attenuated HRV and an increased low‐to‐high frequency ratio, indicative of heightened sympathetic tone and diminished parasympathetic activity. Conversely, studies employing sympathetic skin response, tilt testing, and skin conductance tests have produced more variable results, with only a slight majority supporting the presence of autonomic dysfunction in FM [20].

In this context, nerve US has emerged as an increasingly valuable complementary tool for diagnosing polyneuropathies by identifying morphological changes such as increased CSA. Significant CSA increases have been observed in various immune‐mediated polyneuropathies, such as chronic inflammatory demyelinating polyneuropathy (CIDP) and hereditary neuropathies, each displaying distinct characteristics [21]. Recent studies have demonstrated the utility of US pattern sum‐score (UPSS) in distinguishing between demyelinating and axonal neuropathies, with CIDP patients showing significantly higher scores [22]. Moreover, power Doppler US has proven particularly useful in detecting moderate‐to‐severe compression neuropathies, such as carpal and cubital tunnel syndromes [23].

Overall, while nerve US is not suitable as a standalone screening test, it is a practical, widely accessible, and reproducible diagnostic tool that enhances the evaluation and management of peripheral neuropathies. Key parameters to assess include CSA, echogenicity, morphology, epineurium thickness, vascularization, and nerve mobility [12].

Our previous US study identified an increased CSA of the sural nerve in FM patients, suggesting underlying peripheral nerve involvement. This study demonstrated a significant relationship between the sural nerve CSA and BMI, indicating that higher BMI may be associated with more pronounced peripheral alterations in FM. Additionally, the CSA correlated with neuropathic pain features assessed using the Pain Detect Questionnaire (PDQ), highlighting an association between CSA and symptom severity [24].

Subsequent research conducted at our center focused on morphological changes in multiple peripheral nerves using high‐resolution US. This study evaluated several nerves included in the UPSS, specifically the vagus nerve, C6 nerve root, ulnar nerve at two levels, median nerve at three levels, tibial nerve at two levels, fibular nerve, and sural nerve. Findings revealed significantly increased CSA in FM patients at the vagus nerve, sural nerve, and C6 nerve root compared to healthy controls (*p* < 0.001 for all sites). This enlargement may reflect peripheral nerve pathology contributing to central sensitization in FM. However, clinical correlations between increased CSA and variables such as disease severity, neuropathic pain features, depression, anxiety, and fatigue were weak, underscoring the complex relationship between structural changes and symptomatology in FM [14].

Although no other studies have specifically investigated sural nerve CSA via US in FM, our findings are consistent with research on SFN and nerve US. Ebadi et al. reported that patients with SFN exhibited an enlarged sural nerve CSA compared to healthy controls, without significant differences in nerve echogenicity [13].

Similarly, studies on Sjögren's syndrome‐associated peripheral neuropathy detected significant sural nerve enlargement, with a CSA cutoff of 2.6 mm^2^ effectively differentiating SFN patients from controls. Some patients with normal IENFD but symptoms of small fiber dysfunction exhibited enlarged sural nerve CSA, suggesting small fiber involvement despite preserved large fiber function [25].

The precise mechanisms underlying larger nerve enlargement remain unclear. Small‐caliber nerve fibers affected in SFN are terminal branches of larger nerves, such as the sural nerve. It is hypothesized that distal nerve fiber loss disrupts axoplasmic flow in proximal nerve segments, leading to enlargement. Other potential mechanisms include sodium channel dysfunction, as observed in polyneuropathies like diabetic sensorimotor neuropathy, or CD4+ T lymphocyte infiltration contributing to nerve swelling [13, 26]. In contrast, advanced uremic neuropathy demonstrates reduced sural nerve CSA, reflecting associated axonal loss in severe conditions [27].

Regarding the vagus nerve, while our study found increased CSA in FM patients, another study exploring autonomic dysfunction in FM and generalized anxiety disorder reported reduced vagus nerve CSA in both conditions. The similarity between these groups suggests shared autonomic nervous system abnormalities, potentially through vagus nerve atrophy.

To our knowledge, this is the first study using US imaging to longitudinally investigate peripheral nerve alterations in FM. Over a 24‐month follow‐up, FM patients exhibited persistent sural and vagus nerve CSA enlargement compared to controls, supporting the hypothesis that SFN and autonomic dysfunction are integral to FM. However, there were no statistically significant changes in nerve size over time, suggesting that neuropathy may remain stable rather than progressive during this period.

These findings align with Quitadamo et al., who observed stable IENFD and clinical features over an 18‐month follow‐up in FM patients using skin biopsy. Notably, reduced IENFD was linked to greater disability progression, suggesting that small fiber dysfunction may influence FM evolution, particularly motor performance [28]. Unlike isolated SFN, which often improves, nerve impairment in FM appears persistent rather than progressive [29].

Comprehensive clinical assessments, including the FIQR, DN4, COMPASS‐31, WPI, and SSS, provided insights into FM severity. Interestingly, patients with higher clinimetric scores did not exhibit increased sural or vagus nerve size, with some exceptions. Significant changes in vagus nerve size were observed in patients with neuropathic pain features, reflected in higher DN4 scores. Additionally, baseline autonomic dysfunction, as indicated by higher COMPASS‐31 scores, was associated with greater sural CSA values. These results suggest that peripheral nerve changes may represent only one aspect of FM and do not consistently reflect disease severity.

The main limitations of this study include the lack of histological examinations, considered the gold standard for SFN diagnosis, and a relatively small, single‐center patient cohort. Future studies should correlate US findings with histological data and evaluate nerve echogenicity in FM patients compared to healthy controls. An additional limitation is the lack of a systematic electrophysiological assessment, which prevents us from definitively excluding the possibility of asymptomatic large fiber involvement in some patients.

In conclusion, US assessment of peripheral nerves, particularly the sural and vagus nerves is a valuable, noninvasive tool for detecting peripheral nerve involvement in FM. At these two peripheral sites, US is practical, reproducible, widely accessible, and minimally contraindicated, offering insights into FM's underlying mechanisms. Persistent nerve enlargement in some FM patients suggests a potential subgroup with significant peripheral involvement, which could provide information about the physiopathological pathway and therapeutic strategies. Validation through larger, multicenter studies incorporating histological data is essential to confirm these findings and establish nerve US as an imaging biomarker for FM.

## Conflicts of Interest

The authors declare no conflicts of interest.

## Data Availability

Data are available upon reasonable request to the corresponding author.
